# Leaky Gut and Autoimmunity: An Intricate Balance in Individuals Health and the Diseased State

**DOI:** 10.3390/ijms21249770

**Published:** 2020-12-21

**Authors:** Bilal Ahmad Paray, Mohammed Fahad Albeshr, Arif Tasleem Jan, Irfan A. Rather

**Affiliations:** 1 Department of Zoology, College of Science, King Saud University, P.O. Box 2455, Riyadh 11451, Saudi Arabia; bparay@ksu.edu.sa (B.A.P.); albeshr@ksu.edu.sa (M.F.A.); 2 School of Biosciences and Biotechnology, Baba Ghulam Shah Badshah University, Rajouri 185234, India; 3 Department of Biological Science, Faculty of Science, King Abdulaziz University, Jeddah 21589, Saudi Arabia

**Keywords:** autoimmune disorders, immune system, intestinal wall, microbiota

## Abstract

Damage to the tissue and the ruining of functions characterize autoimmune syndromes. This review centers around leaky gut syndromes and how they stimulate autoimmune pathogenesis. Lymphoid tissue commonly associated with the gut, together with the neuroendocrine network, collaborates with the intestinal epithelial wall, with its paracellular tight junctions, to maintain the balance, tolerance, and resistance to foreign/neo-antigens. The physiological regulator of paracellular tight junctions plays a vital role in transferring macromolecules across the intestinal barrier and thereby maintains immune response equilibrium. A new paradigm has explained the intricacies of disease development and proposed that the processes can be prevented if the interaction between the genetic factor and environmental causes is barred by re-instituting the intestinal wall function. The latest clinical evidence and animal models reinforce this current thought and offer the basis for innovative methodologies to thwart and treat autoimmune syndromes.

## 1. Introduction

The gastrointestinal (GI) tract’s epithelium represents the largest mucosal lining that provides an interface between mammalian host and the external environment. The lumen of the gut harboring trillions of microbial inhabitants plays a vital role digestion and influences the immune system. The outstanding anatomical architecture of the GI fine-tunes processes like absorption and digestion of food, neuroendocrine networking, and immunological balance [1]. Despite being in continuous interaction with various foodborne pathogens and antigens, the GI lining very efficiently checks invasion by microorganisms and other molecules through its paracellular space, thereby maintaining its capability of intestinal permeability. The paracellular space size ranges approximately from 10 to 15 Å, implying that, under physiological situations, solutes with a molecular radius above 15 Å (~3.5 kDa) have to be barred from the uptake path [2]. The transfer of macromolecules is controlled principally by epithelia’s paracellular permeability, whose regulation is dependent on the attuned intercellular tight junctions [3]. A fast-rising number of illnesses, including autoimmune diseases, are reported due to intestinal permeability changes relative to changes in tight junctions (TJs) [4]. The change in permeability of the GI tract’s epithelial lining creates an easy passage for commensal bacteria and their products from the lumen into the bloodstream (referred to as the leaky gut), thereby evoking immune response. Studies have well documented autoimmune diseases that arise due to the underlying phenomenal problem of the leaky gut [5,6].

An autoimmune disease occurs when the immune system produces autoantibodies against self-antigens, causing assault on body tissue. An association of autoimmune diseases and leaky gut has emerged as a critical situation wherein the leakage of pathogens into the body system results in autoimmunity [6]. As such, maintenance of the healthy gut goes a long way in preventing autoimmune diseases. This review focuses on the relationship between the leaky gut and autoimmune diseases, causes of leaky gut, factors contributing to the healing of leaky gut, and a prospectus of ongoing research being carried out on the topic.

## 2. Intestinal Barrier Regulation

**Figure 1:**
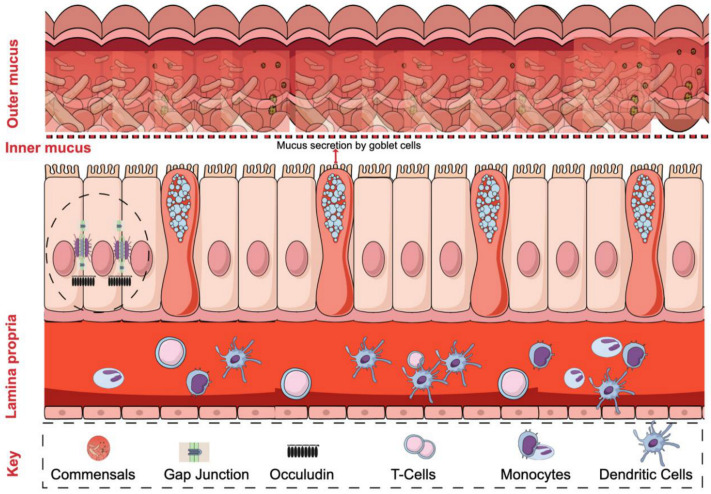
Schematic of the intestinal epithelial barrier. The lining of epithelial cells forms a barrier between the host body and the gut’s lumen with commensal bacteria. The functional barrier cells are held together by many tight junction proteins that hinder the entry of bacteria. The mucosal layer acts as a first-line defense that prevents the contact of gut microbiome with intestinal epithelial cells. The role of immune cells against infection further maintains the hemostasis of the intestinal epithelial barrier.

The single layer of epithelium in the intestinal wall acts as a barrier that separates the host body from the external environment. The regulation of a functional barrier is maintained by intestinal epithelial cells (IECs). The lining of IECs shows unique intercellular connections displaying TJs, desmosomes, and adherent junctions that allow nutrient and fluid absorption; however, it prevents the passing of unwanted microbes or antigens to the underlying tissue [7,8]. TJs are conceptualized as a dynamic structural barrier in the paracellular space [7], with protein composition that includes a major proportion of claudins, occluding, and junctional adhesion molecules (JAMs) [8]. Besides, the lamina propria of the intestinal epithelial system underneath the IECs holds a number of immune cells, not limited to T-cells, B-cells, macrophages, and dendritic cells. These cells play an essential role in maintaining intestinal homeostasis (Figure 1). The components of the immune system like tumor necrosis factor-*α* (TNF-*α*), interleukins, and interferon-*γ* are inevitable in the regulation of TJs. TNF-*α* mediated activation of NF-κB is one such important regulator, and studies have shown that the inhibition of the latter leads to protective effects against severe diarrhea [8].

Mucin, a highly glycosylated polymeric protein in the mucous layer, also plays an important role in trapping pathogens thereby preventing microbial colonization. The absence of a mucin layer, or a disturbed mucin layer has been seen to make animals vulnerable to intestinal inflammation [9]. The remarkable study on zonula occludens toxin (Zot) enterotoxin elaborated by *Vibrio cholera* showed that it reversibly opened TJs, which potentiated our knowledge of the regulation of the paracellular pathway [10]. Zonulin, being the eukaryotic counterpart, has been known to regulate TJs in relation to fluid and macromolecular movement and the exchange of leukocytes between the lumen of intestine and the bloodstream (by increasing intestinal permeability). Small intestines exposed to enteric bacteria secreted zonulin [11]. The role of zonulin in displaying innate immunity may represent a defensive mechanism that inhibits microorganisms that colonize the small intestine [11].

## 3. Causes of Leaky Gut

The cause of leaky gut includes prolonged contact with an environmental contaminant, overconsumption of alcoholic beverages, and unhealthy food choices [12]. Mental stress for an extended period inhibits the capacity of the immune system to respond speedily and slows down its ability to heal. The flow of blood to digestive organs is reduced, and there is an increase in the generation of toxic metabolites that cause a permanent deferral of the necessary repair routine [13]. The immune system responds to many places at once, and the parts of the body located far away from the intestinal system are easily affected. 

The vertebrate GI tract comprises an extraordinary chemical composition and a thick microbial atmosphere, which influence the immune reactions of host cells and excite a rich medium of effector mechanisms involved in innate and acquired immune responses. Any perturbations in the structural dynamics of the microbial community and their functions within the intestinal tract (referred to as dysbiosis) also become a cause of leaky gut condition and, ultimately, the occurrence of autoimmune diseases [14]. Microbial inhabitants of the GI tract are unique in shaping the host’s immunity and regulating metabolism [14]. Newly studied data specify a serious role of gut microbiota associated with autoimmune diseases [15,16]. Many *Candida* spp. on reaching the lining of the gastrointestinal wall cause collapse of the brush border epithelium lining the GI tract [17]. In addition, *Salmonella* sp., *Giardia* sp., *Yersinia* sp., *Helicobacter pylori*, *Blastocystis hominids*, *Shigella* sp., and other pathogenic microbes disrupt the intestinal lining, thereby causing gastrointestinal problems [18]. The digestive diseases or liver damage cases have amplified the propensity towards the leaky gut condition. 

Beverages have few nutrients but take several nutrients to metabolize. The most notable of these nutrients are B-complex vitamins [13]. As part of metabolism in the liver, the contaminants are either broken down or stockpiled by the body. The abuse of overconsumption of beverages puts stress on the liver, which upsets the digestive ability and harms the GI tract [19]. Food with little fiber increases transit time, thereby increasing exposure to harmful by-products of digestion that cause irritation of the gut mucosa [20]. Additionally, processed foods contain many additives capable of promoting inflammation of the GI tract [20]. Non-steroidal medicines, aspirin, and Motrin mutilate the brush borders, permitting microbes, food particles that are not wholly digested, and contaminants to go into the bloodstream [13]. Birth control drugs and steroids also form favorable conditions for fungi nourishment, which cause damage to the lining. Chemotherapy and radiation treatments significantly disrupt the balance of the GI wall [21]. Additionally, sensitivity to certain foods and the environment could lead to the development of leaky gut syndrome [22,23].

## 4. Factors Contributing to the Healing of Leaky Gut

In the early 1860s, microbial intoxication of the gut was believed to be a major cause of systemic illnesses and mental disorders, and, for many decades, scientists agreed. Modern studies validate these allegations by illustrating that commensal microflora being recognized by Toll-like receptors (TLRs) is essential for promoting epithelial cell multiplication. Hence, it accelerates the healing of the surface epithelium after being damaged and impedes inflammation [24,25]. TLR signaling is critically vital for protection from pathogenic irritation and for promoting tolerant conditions to commensalism [26]. The gut microbiome interacts with the host cells under the influence of a highly regulated immune system that involves pattern recognition receptors (PRRs) like TLRs and NOD-like receptors (NLRs) [27]. The stimulation of TLRs boosts intestinal epithelial integrity through translocation of the tight junction protein zonula occludens-1 (ZO-1) [26,28,29]. A pathogen being acknowledged by specific TLRs results in several events, including the stimulation of NF-κB signaling that follows an increase in the production of cytokine and T-cell activation.

The mucosal structure is known for its natural resistance and capacity to differentiate possibly pathogenic bacteria from inoffensive antigens, which is realized via the arrangement of recognition receptors [26]. The permeability of the intestinal epithelium has also been well known to be modulated by the commensal microbiota and their productions [30]. The reversal of leaky gut through administration of probiotics and prebiotics has gained momentum in the last decade. Reviews confirm the reversal of leaky gut by probiotics through augmentation of TJ protein production [30]. Studies have shown that the fermentation activity of gut microbes exert healing effects not only on the intestinal epithelium integrity but also permeability [31]. Fermentation products have also been shown to modulate anti-inflammatory signals necessary for an adequate active immune response [32]. 

The gut shows diverse microbial communities in different parts. Streptococcaceae and Lactobacillaceae populate the proximal region, whereas the distal portion of the small intestine is home to Lactobacillaceae, Erysipelotrichaceae, and Enterobacteriaceae. The colon is inhabited by members of Bacteriodaceae, Prevotellaceae, and Clostridiaceae families. The epithelial lining of stomach harbors Lactobacillaceae and Streptococcaceae [33]. The term dysbiosis is commonly used to describe the situation that arises whenever there is a structural or functional change in gut microbiota configuration, which disturbs homeostasis of the gut [33]. Fermentation products are also known to become imbalanced under dysbiosis. The infection-destroying segments of microbiota may altogether be countered with inflammation-aggravating germs, improving the wall effect of the gastrointestinal mucosa and straightening the interaction with inflammation-causing constituents of the immune system [34]. As further studies show, regulating the immune system is one way of fixing a leaky gut [35,36]. Dysbiosis upregulates the expression of TLRs on antigen-presenting cells (APCs) and disturbs the T-cell balance [37]. Reports indicate that dysbiosis promotes the production of neo-antigenic determinants of self-proteins, thereby evoking autoimmunity [38].

## 5. Autoimmune Diseases Associated with Leaky Gut

**Figure 2:**
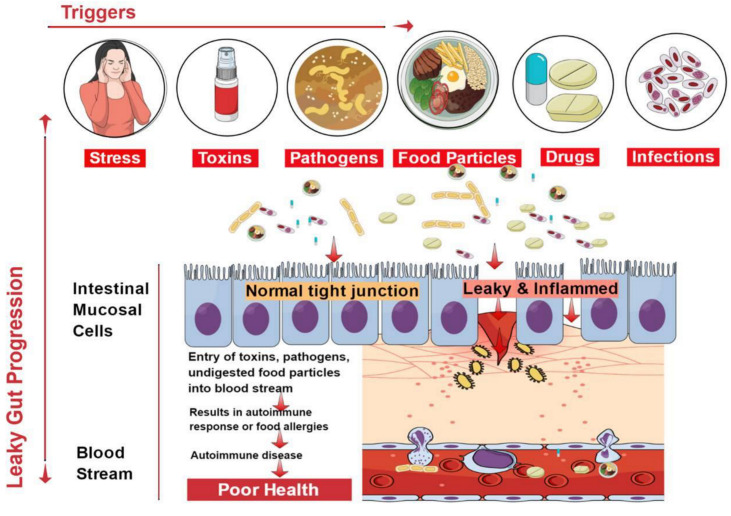
Factors contributing to the development of leaky gut and its relationship to autoimmune diseases. Diet, genetic susceptibility, and environmental conditions, among others, affect the intestinal epithelial barrier integrity. This imbalance leads to compromised barrier integrity and contributes to several diseases.

The epithelial lining of the gastrointestinal mucosa acts as a barrier against the gut luminal content, thereby preventing the passage of elements that can cause harm to the host system [13]. A breach in the epithelial barrier by foreign entities from the lumen into the host sets out a series of events that turns the immune system against the host itself, thereby presenting a plethora of autoimmune diseases like type 1 diabetes (T1D), multiple sclerosis (MS), inflammatory bowel disease (IBD), systemic lupus erythematosus (SLE), etc. [4,39] (Figure 2). 

The situation of impaired barrier function in the mucosal lining of the GI tract, which results in even larger holes in the lining, manifests in leaky gut. Thus, things that were initially barred from passing through (e.g., proteins, gluten, microbes, and food antigens) can now breach through the tissue as well as systemic circulation, resulting in intestinal inflammation that may trigger an array of autoimmune diseases such as inflammatory bowel disease, celiac diseases, autoimmune hepatitis, multiple sclerosis, etc. [4,40,41,42,43,44]. We have accumulating evidence in support of the presence of overexpressed zonulin in subjects with autoimmune diseases [41]. Zonulin has been recognized as pre-haptoglobin (HP)2 [45]. The release of zonulin has been implicated in the pathogenesis of autoimmune diseases where the stimuli are bacteria, both the gut commensals and pathogens, and food antigens like gluten [6]. Under the conditions of compromised TJ function, an immune response ensues after antigen stimulation. The immune cells such as antigen-presenting cells (APCs), T-cells, T killer cells, B-cells, and plasma cells in the intestinal barrier get activated [6]. Dysbiosis induced by leaky gut presents an inflammatory environment that paves the way to autoimmunity. Microbial translocation induces pro-inflammatory cytokines such as IFN-*γ*, TNF-*α*, and IL-13 [37].

Herein we present a brief account of some of the known autoimmune diseases due to their connection with leaky gut syndrome with evidence supporting the paradigm that leaky gut causes autoimmunity [46]. 

### 5.1. Type 1 Diabetes 

T1D, an autoimmune disease mediated by self-reactive T-cells, is characterized by the destruction of insulin-producing β-cells in the host’s pancreas [47,48]. GI signs of diabetes mellitus have widely been attributed to the changed intestinal permeability secondary to autonomic neuropathy [49,50]. However, suggestions from other studies indicate that an increase in the permeability of the intestinal tight junctions is accountable for the start of the ailment, and GI symptoms are regularly experienced by these patients [51]. Studies in T1D models have confirmed that changes in the gut wall (of the large intestine) help the luminal bacteria breach into the extraluminal tissues [52]. This hypothesis is reinforced by a clinical study carried out in humans and on a diabetic animal model [53,54,55,56]. In one study, an increase in a rat’s small intestine permeability preceded the onset of diabetes by no less than a month [57]. Reports show that increased local permeability of the intestinal mucosa showed an increase in proinflammatory cytokines leading to a deranged immune system [49]. In both human and animal studies, gliadin, acting as an antigen, is a trigging factor connected to the autoimmunity of T1D [58].

Studies have attested that the pathogenesis of T1D displays an altered gut microbiome and, hence, dysbiosis [27]. Data support that gut bacteria can profoundly affect the prevention of autoimmune diabetes [27]. Leaky gut induced dysbiosis causes translocation of gut bacteria into pancreatic lymph nodes, whereby induction of pathogenic T helper cells contributes to T1D [27]. Gut microflora was found to protect against the development of T1D in two animal models, bio-breeding disease-prone (BB-DP) rats, and non-obese diabetic (NOD) mice [59,60]. Moreover, GI microbiota changes [61] and the use of zonulin inhibitors were found to ameliorate the manifestation of T1D in rat models of the disease [11,57,62]. 

### 5.2. Multiple Sclerosis

Apart from an increase in the permeability of the blood–brain barrier, subjects with multiple sclerosis were found to exhibit an increased permeability at intestinal tight junctions [7]. A quarter of patients with multiple sclerosis had an increased permeability of the intestinal walls [40]. Multiple sclerosis and Crohn’s disease patients exhibit an increase in the number of peripheral B cells, a sign of antigen exposure. This further reinforces the notion of preexisting, genetic abnormalities in the permeability of the small intestine, with a consequently changed antigen exposure as a pathogenic issue common to the two diseases [40]. Bacterial infections are believed to cause multiple sclerosis, though clear epidemiological evidence is lacking. This supports the notion that commensal bacteria contribute to MS pathogenesis, and the effects of nutrition on MS advancements provide various forms of indirect evidence [63,64]. Experimental autoimmune encephalomyelitis (EAE), an animal model of MS, suggests that the gut flora contributes to the development of this illness, and therapy involves administration of probiotics (i.e., live beneficial bacteria) or prebiotics (i.e., compounds that stimulate the growth of beneficial bacteria) [65]. EAE is usually induced in experimental animals by immunization with myelin antigens in a blend with a potent adjuvant [63]. In contrast, stomach treatment with a combination of antibiotics reduced the severity of EAE [63]. Attenuation of the pro-inflammatory TH1/TH17 responses helps in reducing the impact of the demyelinating syndrome. Lee and colleagues demonstrated that disease defense in germ-free mice matched with lowered levels of the pro-inflammatory cytokines IL-17 and IFN-c and raised numbers of Forkhead box P3+ (Foxp3+) regulatory T (Treg) cells in peripheral lymphoid tissues and the CNS [66]. Moreover, IL-10-producing, Foxp3+ Treg cells, which accumulate in the cervical LNs (cLNs) of antibiotic-treated mice, could protect innocent recipients from the transfer of EAE. We have accumulating evidence in support of dysbiosis in MS [67].

### 5.3. Inflammatory Bowel Diseases

IBD, which involves Crohn’s disease (CD) and ulcerative colitis (UC), occurs due to defects in the paracellular permeability of the GI tract [68]. The pathogenesis of IBD is multifactorial. In recent years, substantial evidence has supported the theory that environmental, genetic, and immunological factors instigate the autoimmune course [69]. Despite this, most evidence suggests the contribution of increased gastric permeability to the pathogenesis of IBD [70]. In Crohn’s disease, an increase in the permeability of the intestinal epithelial comes before clinical relapse, which suggests that a defect in permeability is the first event in the occurrence of the disease [71,72]. Reports suggest that during the development of IBD, a defective intestinal barrier displays the expression of cytokines, IFN-*γ,* and TNF-*α,* thereby initiating inflammation [73]. Paradoxically, this presents a situation wherein the deranged immune system in the intestinal tissue triggers further leaks in the luminal wall. The condition of dysbiosis plays an important role in the pathogenesis of IBD [74]. The dysbiosis in IBD is seen as an imbalance arising in the densities of obligate and facultative anaerobes that result in the pathogenesis of IBD [75].

### 5.4. Ankylosing Spondylitis

Ankylosing spondylitis (AS) is a common rheumatic syndrome that distinctively affects young adults. It is characterized by a stiff and painful back [76]. The connection between an increase in the permeability of the intestines and the syndrome has been plainly established [77]. Using a proteomic method, an investigation of the serum protein summaries of AS patients and healthy controls from a Chinese AS family has been conducted. A group of four massively expressed protein spots was clearly witnessed in every AS patient’s summary and consequently recognized as isoforms of HP [69]. The role of dysbiosis in AS has been demonstrated by showing the active participation of ileal bacteria in modulating local and systemic immune responses [78]. The gut vasculature showed impairment that caused a significant rise in zonulin levels, which affected the TJs.

### 5.5. Systemic Lupus Erythematosus

SLE, a prototypical multisystem autoimmune disease characterized by a hyperactive immune response that causes severe and persistent inflammation, often leads to multiorganelle damage [79]. Though the etiology of SLE is unclear, various genetic and environmental factors are involved in the occurrence of the disease [80]. Increased bacterial lipopolysaccharide (LPS) uptake via the gut lumen promotes the development and progression of SLE [5]. The addition of TLR4 activation, with LPS inducing the release of CD14 from monocytes, exacerbates SLE development [24,25,81]. Dysbiosis induced by leaky gut enhances inflammatory macrophagic activity that damages the tissues in SLE [27]. Enhancement in the commensal gut microbiota through supplementation of probiotics significantly ameliorates the occurrence of the disease [79,82,83]. 

### 5.6. Healing the Leaky Gut

Many studies have revealed that a leaky gut paves the way to the development of autoimmune diseases. Therefore, healing the leaky gut suppresses the symptoms of these diseases; as such, decreasing its occurrence is vital to the prevention of autoimmune diseases [84,85]. The process of healing the gut has also been looked into, with both short-term and long-term measures. Short-term measures to heal the gut include discontinuing foods rich in gluten, dairy, and sugar from the diet [86]. Additionally, raw foods eaten in moderation and consumption of tea and bone broth are vital to healing [87]. In the long term, maintaining good gut health is crucial to the prevention of autoimmune diseases by sustaining proper gut health. Similarly, terraforming is a long-term method of preventing leaky gut. Furthermore, the addition of prebiotics helps in establishing the gut flora by creating a fresh system of operation in the gut [87]. Thus, the inclusion of probiotics and prebiotics in the daily diet can augment gut microbiome health by reducing intestinal permeability [5].

Treatment with *Bacteroides fragilis* has been shown to decrease the pathogen translocation that further ameliorates the diseased state [5]. The role of *B. fragilis* has been predominantly shown to change the microbial flora and improve the barrier function of the intestine. As most autoimmune diseases involve the imbalance of the microbiota and hyperactivity of the immune system, systemic immune modulation through an extraneous supply of substances that either supplement internal microbiota or promote their proliferation is possible [88,89]. One such measure to increase resident microflora has been achieved using probiotics and prebiotics [89]. Probiotics (i.e., healthy microflora) and prebiotics (i.e., food compounds promoting the proliferation of probiotics) improve the gut environment on administration, prevent the colonization of pathogenic microbes, and regulate immune function. They reduce the permeability of the gut lining and, as such, confer health benefits to the host. The introduction of probiotics (most of the lactobacilli) modulates the gut microbiota and decreases the occurrence of autoimmune diseases such as IBD and T1D [89,90]. The most common prebiotic supplement derived from plants is inulin. The supplementation of diet with inulin enhances the growth of *Bifidobacterium* spp., besides improving glucose homeostasis [91,92].

## 6. Conclusions

Autoimmune disorders are facilitated by heredity, the environment, contaminants, and altered gut microbiota. Acting as fueling forces in the facilitation of autoimmune disorders, there has been fantastic advancement in our understanding of the interplay among these factors. Genetic predisposition, exposure to triggering environmental factors, and damage to intestinal wall function, secondary to poor functioning of paracellular tight junctions, appear to be crucial ingredients presented in the pathogenesis of autoimmune diseases. The traditional model of autoimmune pathogenesis relating to a particular genetic constitution and contact with environmental triggers has lately been challenged by the inclusion of a third component: damaged GI function. In T1D, gliadin can contribute to the loss of stomach wall function and can prompt the autoimmune reaction in genetically prone persons. This recent theory suggests that as soon as the autoimmune process is triggered, it is not auto-continuing, but can rather be moderated or even overturned by inhibiting the constant interplay between the genetic factor and the environment. Since tight junctions are components of reduced functioning in this interaction, new healing approaches are aimed at reinventing the gut barrier function, providing inventive, unexplored methods for the treatment of these cataclysmic diseases. We emphasize on more studies involving the application of probiotics that can show the reversal of dysbiosis aiming at disease attenuation. Keeping in view of the cost-effectiveness of these treatment modalities, autoimmune diseases in coherence with leaky gut can be well handled, and we sincerely foresee a future approach to focus on more of such studies.
